# The efficacy and safety of reducing intraocular pressure to single‐digit levels in Normal‐tension glaucoma: A systematic review and meta‐analysis

**DOI:** 10.1111/aos.70110

**Published:** 2026-03-20

**Authors:** Beatriz Araújo‐Azevedo, Renato Ferreira‐da‐Silva, João Barbosa‐Breda

**Affiliations:** ^1^ Faculty of Medicine of the University of Porto Porto Portugal; ^2^ Porto Pharmacovigilance Centre Faculty of Medicine of the University of Porto Porto Portugal; ^3^ RISE‐Health, Department of Community Medicine, Information and Health Decision Sciences Faculty of Medicine of the University of Porto Porto Portugal; ^4^ RISE‐Health, Department of Surgery and Physiology Faculty of Medicine of the University of Porto; ^5^ Department of Ophthalmology Local Health Unit of São João Porto Portugal; ^6^ Department of Neurosciences KULeuven, Research Group Ophthalmology Leuven Belgium

**Keywords:** glaucoma progression, intraocular pressure, low tension glaucoma, Normal tension glaucoma, systematic review

## Abstract

**Purpose:**

This systematic review and meta‐analysis aim to evaluate the efficacy and safety of further lowering intraocular pressure (IOP) to single‐digit levels in treated patients with progressive normal‐tension glaucoma (NTG).

**Methods:**

A literature search was conducted in MEDLINE (via PubMed), Scopus and Web of Science. We selected studies that reported quantitative visual field (VF) data on the efficacy of the interventions. Study outcomes include VF progression rates, changes in IOP and IOP‐lowering medication use. Meta‐analysis was conducted for VF mean deviation (MD) slope (db/year) using R, with paired mean differences and 95% confidence intervals (CI) calculated.

**Results:**

Nine observational studies, including 270 eyes, were analysed. Trabeculectomy was performed across all studies. One study compared trabeculectomy with prostaglandin analogues. On average, IOP decreased from 14.77 ± 0.95 mmHg preoperatively to 9.82 ± 1.55 mmHg postoperatively, reflecting a mean reduction of 33.65 ± 10.37%. This reduction slowed glaucoma progression, as indicated by a change in the mean VF MD slope from −0.91 ± 0.27 dB/year preoperatively to −0.25 ± 0.16 dB/year postoperatively. Meta‐analysis showed a significant change in VF MD slope following trabeculectomy, with a pooled mean difference of 0.59 dB/year (95% CI: 0.38 to 0.80; *p* < 0.0001). Single‐digit IOP levels were shown to be both achievable and sustainable. The most common postoperative complications included hypotony‐related complications and hyphema.

**Conclusions:**

Achieving single‐digit IOP levels is associated with a significant reduction in visual field progression rates. However, the balance between efficacy and safety must be considered, as aggressive IOP reduction is also associated with a higher risk of hypotony‐related complications. Postoperative hypotony was relatively frequent but generally transient and did not appear to have a significant impact on long‐term visual acuity. Copyright © 2026 Acta Ophthalmologica Scandinavica Foundation.

## 1 INTRODUCTION

Glaucoma is a chronic, progressive condition characterized by both structural and functional alterations of the optic nerve. Although elevated intraocular pressure (IOP) is the primary modifiable risk factor influencing the onset and progression of the disease, glaucoma can still develop even when IOP remains within the statistically defined normal range (Group, 1998). Normal‐tension glaucoma (NTG) is a progressive optic neuropathy in which IOP remains within the normal range (≤21 mmHg, below the upper statistical threshold of the normal range) (Killer & Pircher, 2018). The prevalence of NTG varies considerably worldwide, and in some East Asian populations, it accounts for the vast majority of open‐angle glaucoma cases. For instance, the Tajimi Study reported that up to 92% of primary open‐angle glaucoma (POAG) cases in Japan were classified as NTG (Iwase et al., 2004).

Although IOP remains within the normal range in NTG, optic disc excavation and visual field (VF) defects can still be observed (Cho & Kee, 2014). This indicates a multifactorial aetiology, where IOP‐independent mechanisms contribute to the pathogenesis of NTG. Furthermore, NTG eyes may have different levels of tolerance and/or genetic predisposition to an IOP within the normal range, depending on demographic factors (Chon et al., 2013). Given its complex aetiology, NTG is a diagnostic challenge. Early screening and diagnosis are crucial for early detection and intervention, as the disease often remains asymptomatic until reaching advanced stages (Kim & Park, 2016).

The primary goal of glaucoma treatment is to prevent disease onset and progression while preserving visual function throughout the patient's lifetime (Chen, 2020). IOP reduction remains the cornerstone of treatment (Killer & Pircher, 2018). However, despite optimized medical therapy, some NTG patients continue to experience disease progression even after IOP has been lowered to the low teens. These cases present a significant therapeutic challenge, often requiring further IOP reduction—potentially to single‐digit levels—to preserve the remaining VF and prevent further deterioration (Aoyama et al., 2010; Daugeliene, 1998; Investigators, 2000; Iverson et al., 2016; Koseki et al., 1997; Mataki et al., 2014; Naito et al., 2017; Nakajima et al., 2021; Oie et al., 2017; Shigeeda et al., 2002). The decision to aggressively reduce IOP must be carefully considered, and patient selection should be done with consideration, as this approach may be associated with significant postoperative complications, particularly those related to hypotony. In certain cases, these complications may have a significant impact on patients' visual acuity, despite some studies showing no statistically significant differences in postoperative logMAR BCVA or in the proportion of eyes losing two or more Snellen lines when comparing eyes with hypotony to those without (Meyer et al., 2019; Schultz et al., 2016). These findings suggest that, in selected patients, the functional visual impact of hypotony may be limited, underscoring the importance of balancing the risks and benefits of aggressive IOP lowering strategies.

There is currently no systematic summary of the published evidence evaluating the effects of achieving single‐digit IOP levels in patients with progressive NTG. With that in mind, our systematic review aims to assess the efficacy and safety of achieving single‐digit IOP levels in patients with progressive NTG.

## 2 MATERIALS AND METHODS

### 2.1 Search strategy

We followed the Preferred Reporting Items for Systematic Reviews and Meta‐analyses (PRISMA) checklist. Two authors (JB, BA) independently performed study selection, data extraction and risk‐of‐bias assessments. We conducted a literature search in MEDLINE (via PubMed), Scopus and Web of Science for relevant studies, up to April 29th, 2024. The search was based on the following keywords: ‘Low pressure glaucoma’, ‘Low tension glaucoma’, ‘Normotensive glaucoma’, ‘Normal tension glaucoma’, ‘Normal pressure glaucoma’, “single digit IOP’, ‘IOP < 10’, ‘IOP≤10’, Progression, Progressive (for full queries refer to **Supplemental Content** **1**).

### 2.2 Study selection, data collection process and study outcomes

After the removal of duplicates, the titles and abstracts of the studies identified during the initial search were screened for relevance. Selected studies were then subjected to a further assessment based on predetermined eligibility criteria. Additionally, a hand search was conducted through the reference lists of all included studies to identify additional relevant studies.

We selected the studies based on the following inclusion criteria: (1) observational studies (including prospective or retrospective case series or cohorts) or randomized controlled trials (RCTs), published in any language; (2) studies involving participants diagnosed with NTG; (3) studies that included participants who underwent an intervention aiming for further reduction of IOP to single‐digit or near single‐digit levels; (4) studies providing pre‐ and post‐intervention data on visual field mean deviation (VF MD) slope, allowing for an assessment of the efficacy of lowering IOP to single‐digit targets (some studies considered single digit as ≤10 mmHg).

When available, the data extracted from the selected studies included study characteristics (title, PMID, first author, study design, publication year, sample size, type of procedure and follow‐up duration) and clinical characteristics (glaucoma type, IOP before and after intervention, rate of IOP reduction, mean number of antiglaucoma medications before and after intervention, VF MD slope before and after intervention, visual acuity (VA) measures and main complications after the intervention). For studies that reported visual outcome data at multiple follow‐up time points, the data from the longest follow‐up period were extracted as the post‐intervention data.

### 2.3 Risk‐of‐bias (quality) assessment

Risk of bias was assessed independently by two authors (JB, BA) for each study, using the National Heart, Lung and Blood Institute Study Quality Assessment Tool (see figure, **Supplemental Content** **2**, which demonstrates the tool used).

### 2.4 Quantitative synthesis of results

A meta‐analysis was conducted for a continuous outcome—VF MD slope—reported in its original unit (dB/year). This variable was consistently measured before and after the intervention in the same group of patients. The effect size was calculated as a paired mean difference, reflecting the absolute change following the intervention.

Given the within‐subject nature of the measurements, the calculation of the standard deviation (SD) of the paired differences required an estimate of the correlation between pre‐ and post‐intervention values. In the absence of reported correlations or other statistics that would allow their derivation (e.g. SD of the change, paired t‐tests, or confidence intervals for the change), a moderate correlation coefficient of r = 0.5 was assumed, consistent with the literature. To ensure the robustness of the findings, sensitivity analyses were conducted using alternative plausible values for the correlation (r = 0.25 and r = 0.75), in line with recommendations from the *Cochrane Handbook for Systematic Reviews of Interventions (Section 6.5.2.8)*.

Given the expected clinical and methodological heterogeneity across observational before‐and‐after studies, the meta‐analysis was conducted using a random‐effects model. Between‐study heterogeneity was quantified using the I^2^ statistic and the τ^2^ parameter, with the latter estimated using the Restricted Maximum Likelihood (REML) method. Statistical heterogeneity was assessed through the Q‐Cochran test and the I^2^ statistic, with thresholds of I^2^ > 40% and p‐values < 0.10 used to define substantial and statistically significant heterogeneity, respectively. In cases where substantial heterogeneity was detected, a leave‐one‐out sensitivity analysis was performed to explore the influence of individual studies on the overall pooled estimate.

Potential publication bias was examined visually using funnel plots, and the summary results of the meta‐analysis were graphically represented using forest plots. All statistical analyses were conducted using R software (version 2023.06.1 + 524, R Foundation for Statistical Computing, Vienna, Austria) with the meta and metafor packages.

## 3 RESULTS

### 3.1 Description of studies

Initially, 702 studies were identified across three databases, and five additional records were found through hand‐searching. Duplicates were subsequently removed, resulting in a total of 489 studies. These studies underwent title and abstract screening, leading to the selection of 19 references. A full‐text review was then conducted, and nine articles were considered eligible for inclusion in the final analysis. All included studies were observational in design, comprising eight retrospective cohorts (Aoyama et al., 2010; Daugeliene, 1998; Iverson et al., 2016; Mataki et al., 2014; Naito et al., 2017; Nakajima et al., 2021; Oie et al., 2017; Shigeeda et al., 2002) and one prospective cohort (Koseki et al., 1997). The PRISMA flowchart for this study is presented in Figure 1. Through quality assessment, all nine studies were considered good and, as such, suitable to be included in our systematic review.

**FIGURE 1:**
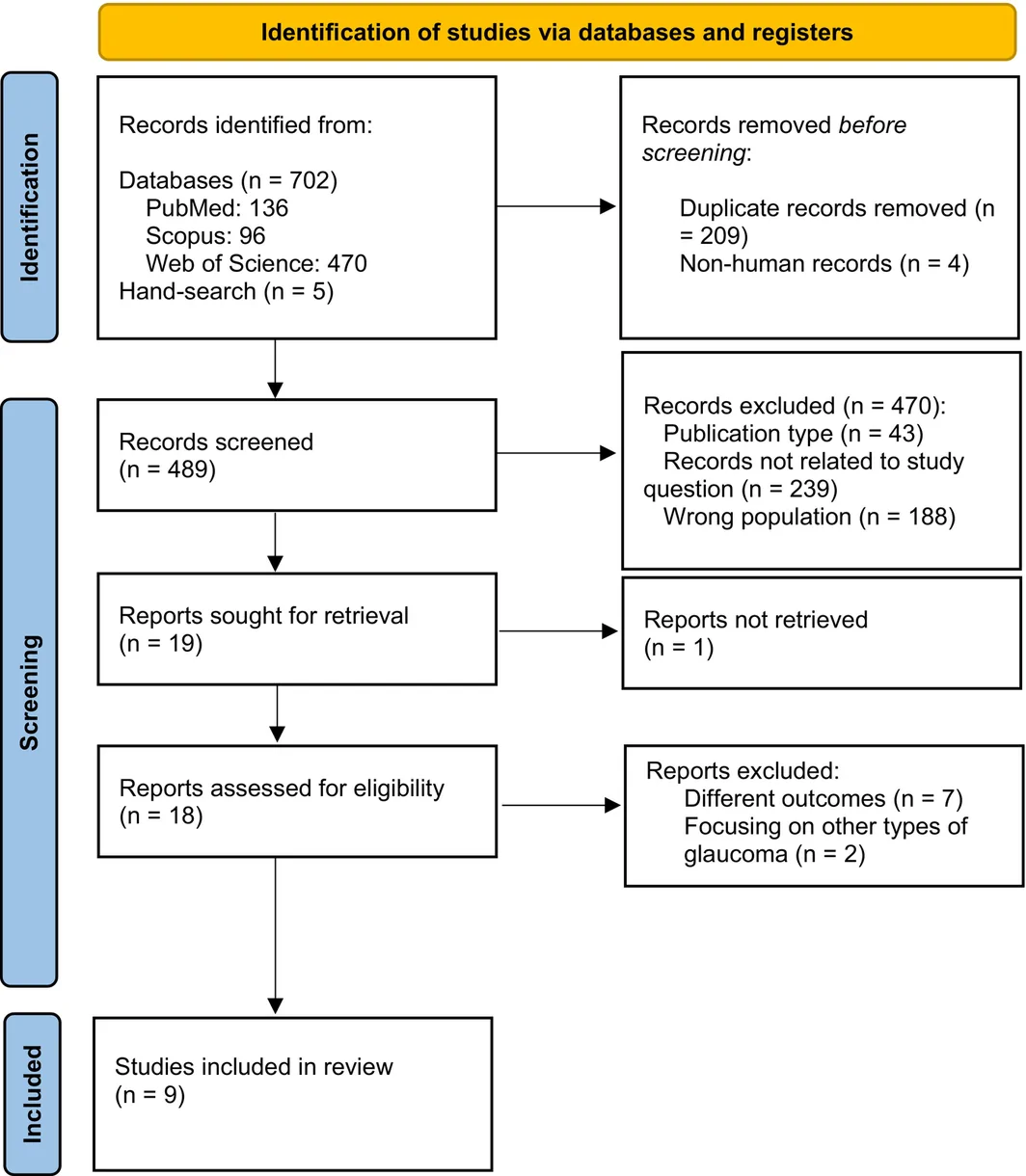
Summary of search and articles selection according to the PRISMA 2020 flow diagram for systematic reviews.

All included studies enrolled patients with progressive NTG undergoing procedures (medical or surgical) to further reduce IOP, resulting in a total of 270 eyes from 266 patients. Of the nine records included, eight were retrospective, and one was prospective. The demographic and clinical characteristics of the included studies were analysed, when available, to determine the overall mean values. The mean age across studies was 60.97 ± 5.44 years. Among studies reporting sex distribution, 147 participants were female (66.2%) and 75 were male (33.8%). The post‐intervention follow‐up period ranged from 37 ± 6 months to 15.6 ± 3.2 years, with a mean follow‐up duration of 7.41 ± 3.7 years. For all eyes, the mean pre‐intervention IOP was 14.77 ± 0.95 mmHg, decreasing to a mean post‐intervention IOP of 9.82 ± 1.55 mmHg, corresponding to an overall IOP reduction of 33.65 ± 10.37% (range: 8% to 42.50 ± 15.80%). The mean VF MD slope also reduced from −0.91 ± 0.27 dB/year to −0.25 ± 0.16 dB/year. In the nine studies included, trabeculectomy with an antimetabolite was the selected intervention to further reduce IOP. In one study, 18 patients underwent trabeculectomy, while 10 patients received phaco‐trabeculectomy (Nakajima et al., 2021). In another study, trabeculectomy was compared to medical treatment with prostaglandin analogues (Oie et al., 2017). Mitomycin C (MMC) and 5‐fluorouracil (5‐FU) were the antimetabolites used across the studies. MMC alone was used in five studies, while four studies used both MMC and 5‐FU, depending on patient characteristics. MMC was applied in concentrations ranging from 0.04% to 0.05% for durations between 1 and 5 min. The concentration of 5‐FU was specified in only one study, which used 50 mg/mL for 5 min. The characteristics of the studies are presented in Table 1.

**TABLE 1:** Characteristics of included studies.

Study	Study design	Sample size (N° eyes)	Age (years, mean ± SD)	Sex (M/F)	Type of glaucoma	Type of procedure	Follow up (mean ± SD)	Baseline IOP (mmHg, mean ± SD)	Baseline IOP lowering medication (n, mean ± SD)
Aoyama et al. (9) 2010	Retrospective	40	56.2 ± 8.5	14/26	NTG	Trabeculectomy	12 ± 2.5 years	15.2 ± 1.4	N/A
DAUGELIENE ET AL. (10) 1998	Retrospective	32	56.9 ± 9.75	15/17	NTG	Trabeculectomy	4.5 ± 1.1 years	14.7 ± 1.6	1.5 ± 0.7
Iverson et al. (11) 2016	Retrospective	15	71.8 ± 7.5	4/11	NTG	Trabeculectomy	68.5 ± 27.8 months	13.11 ± 1.47	2.53 ± 1.25
Koseki et al. (12) 1996	Prospective	21	60.0 ± 10.0	N/A	NTG	Trabeculectomy	37 ± 6 months	16.0 ± 2.1	N/A
Mataki et al. (13) 2014	Retrospective	34	57.6 ± 9.6	12/22	NTG	Trabeculectomy	5.7 ± 1.2 years	15.7 ± 1.7	N/A
Naito et al. (14) 2017	Retrospective	17	69.5 ± 7.6	6/7	NTG	Trabeculectomy	5.0 ± 1.3 years	13.9 ± 0.9	3.0 ± 0.4
Nakajima et al. (15) 2021	Retrospective	28	57.9 ± 10.1	6/22	NTG	Trabeculectomy/phaco‐trabeculectomy	6.0 ± 0.61 years	13.9 ± 3.1	2.64 ± 0.54
Oie et al. (16) 2016	Retrospective	60 (17 + 24 + 19)	55.8 ± 7.9 (Group 1) 63.8 ± 8.7 (Group 2) 58.4 ± 11.8 (Group 3)	7/10 (Group 1) 8/16 (Group 2) 3/16 (Group 3)	NTG	Trabeculectomy/ Prostaglandin analogues	15.6 ± 3.2 years (group 1) 9.8 ± 2.0 years (group 2) 8.1 ± 2.9 years (group 3)	14.7 ± 1.3 (group 1) 14.7 ± 1.5 (group 2) 14.4 ± 1.8 (group 3)	0.9 ± 0.3 (Group 1) 0.3 ± 0.3 (Group 2) 0.3 ± 0.4 (Group 3)
Shigeeda et al. (17) 2002	Retrospective	23	62.8 ± 10.3	N/A	NTG	Trabeculectomy	72 ± 20 months	16.2 ± 1.8	N/A

Abbreviations: F, female; IOP, intraocular pressure; M, male; N/A, not applicable; NTG, normal tension glaucoma; SD, standard deviation.

### 3.2 Does it slow glaucoma progression to further reduce the IOP to single digits?

All studies reported results regarding the efficacy of IOP reduction in VF progression. Based on the studies analysed (Table 2), reducing IOP to single‐digit values was associated with a slowdown in NTG progression, as measured by parameters such as changes in the VF MD slope.

**TABLE 2:** Clinical and visual field parameters before and after intervention.

Study	Baseline IOP (mmHg, mean ± SD)	Final mean IOP (mmHg, mean ± SD)	IOP reduction rate (%)	Baseline IOP lowering medication (n, mean ± SD)	Final IOP lowering medication (n, mean ± SD)	VF MD slope before intervention (dB/year, mean ± SD)	VF MD slope after intervention (dB/year, mean ± SD)	VF defect aggravation (based on the VF MD slope)
Aoyama et al. (9)	15.2 ± 1.4	9.4	38,2	N/A	N/A	N/A	N/A	20.6% for postoperative IOP≤12 50% for postoperative IOP > 12 17.2% for postoperative IOP≤11 45.4% for postoperative IOP > 11 8.3% for postoperative IOP≤10 50.0% for postoperative IOP > 10 11.8% for postoperative IOP≤9 34.8% for postoperative IOP >9
Daugeliene et al. (10)	14.7 ± 1.6	8.7 ± 2.4	40.82 ± 12.10	1.5 ± 0.7	N/A	−0.97 ± 1.07	−0.32 ± 0.61	N/A
Iverson et al. (11)	13.11 ± 1.47	8.47 ± 3.46	35.3 ± 15.0	2.53 ± 1.25	0.80 ± 1.32	−1.05 ± 0.66	−0.26 ± 0.86	N/A
Mataki et al. (12)	15.7 ± 1.7	10.3 ± 2.7	37.0 ± 18.4	N/A	N/A	−0.70 ± 0.18	−0.25 ± 0.17	N/A
Koseki et al. (13)	16.0 ± 2.1	9.2 ± 3.2	42.50 ± 15.80	N/A	N/A	−1.48 ± 1.02	+0.13 ± 1.27	N/A
Naito et al. (14)	13.9 ± 0.9	8.1 ± 2.9	41,7 ± 20.5	3.0 ± 0.4	0.8 ± 1.5	30–2 VF test: −0.91 ± 0.57 10–2 VF test: −0.97 ± 0.70	30–2 VF test: −0.32 ± 0.57 10–2 VF test: −0.09 ± 0.37	N/A
Nakajima et al. (15)	13.9 ± 3.1	9.0 ± 4.6	35.3 ± 19.7	2.6 ± 0.5	0.1 ± 0.4	30–2 VF test: −0.52 ± 0.057 10–2 VF test: −1.00 ± 0.13	30–2 VF test: −0.34 ± 0.17 10–2 VF test: −0.41 ± 0.10	N/A
Oie et al. (16)	Group 1: 14.7 ± 1.3 Group 2: 14.7 ± 1.5 Group 3: 14.4 ± 1.8	Group 1: 9.1 ± 2.0 Group 2: 11.7 ± 1.2 Group 3: 13.2 ± 1.6	Group 1: 37.6 ± 14.3 Group 2: 20.2 ± 3.8 Group 3: 8.2 ± 5.2	Group 1: 0.9 ± 0.3 Group 2: 0.3 ± 0.3 Group 3: 0.3 ± 0.4	Group 1: 0.3 ± 0.4 Group 2: 1.7 ± 0.6 Group 3: 1.7 ± 0.7	Group 1: −0.86 ± 0.51 Group 2: −0.52 ± 0.37 Group 3: No differences in the VF MD slope	Group 1: −0.19 ± 0.20 Group 2: −0.31 ± 0.30 Group 3: No differences in the VF MD slope	N/A
Shigeeda et al. (17)	16.2 ± 1.8	10.8 ± 2.2	33.33 ± 7.73	N/A	N/A	−1.05 ± 0.115	−0.44 ± 0.10	N/A

Abbreviations: IOP, intraocular pressure; MD, mean deviation; N/A, not applicable; SD, standard deviation; VF, visual field.

Daugeliene et al. stated an IOP reduction of 40.82 ± 12.10% after trabeculectomy, from a preoperative mean of 14.7 ± 1.6 mmHg to a mean of 8.7 ± 2.4 mmHg postoperatively (Daugeliene, 1998). The marked IOP lowering was accompanied by a change in VF MD slope that changed from a preoperative mean of −0.97 ± 1.07 dB/year to a postoperative mean of −0.32 ± 0.61 dB/year (p‐value = 0.003) (Daugeliene, 1998).

This beneficial association between lowering IOP to single‐digit levels and changs in VF metrics was verified by additional studies. Iverson et al. reported a significant reduction in IOP from 13.11 ± 1.47 mmHg, using a mean of 2.53 ± 1.25 IOP‐lowering medications preoperatively, to a mean IOP of 8.47 ± 3.46 mmHg and 0.80 ± 1.32 IOP‐lowering medications postoperatively (Iverson et al., 2016). This is equivalent to a reduction of 35% from baseline IOP levels. These changes were associated with a change in the VF MD slope, which went from −1.05 ± 0.66 dB/year preoperatively to −0.25 ± 0.86 dB/year postoperatively (p‐value = 0.05) (Iverson et al., 2016).

Koseki et al. described an average IOP of 16.0 ± 2.1 mmHg preoperatively which, after trabeculectomy, was reduced to a mean of 9.2 ± 3.2 mmHg, contributing to an IOP reduction of 42.50 ± 15.80% (Koseki et al., 1997). The VF MD slope was −1.48 ± 1.02 dB/year preoperatively, and it markedly changed to a mean of +0.13 ± 1.27 dB/year for the first 2 years of postoperative follow‐up (p‐value <0.01) (Koseki et al., 1997).

Mataki et al. observed that lowering IOP from a baseline mean of 15.7 ± 1.7 mmHg to a final mean of 10.3 ± 2.7 mmHg, corresponding to an IOP reduction of 37.0 ± 18.4% after trabeculectomy, was associated with a change in the VF MD slope (−0.70 dB/year to −0.25 ± dB/year, p‐value <0.001) (Mataki et al., 2014). The Spearman correlation coefficient was calculated to assess the relationship between VF MD slope values in the preoperative and postoperative periods and the extent of IOP reduction in each eye. The analysis revealed no significant correlation between these variables. Furthermore, a Mann‐Whitney U test was conducted to assess whether a statistically significant difference existed in VF MD slope values between the preoperative and postoperative periods among eyes with a postoperative mean IOP <10 mmHg and those with IOP >11 mmHg. The results indicated no statistically significant difference between these groups (Mataki et al., 2014).

Similarly, Naito et al. demonstrated an approximate 42% reduction in IOP, from a baseline IOP of 13.9 ± 0.9 mmHg to a final mean IOP of 8.1 ± 2.9 mmHg, which was accompanied by a reduction in the number of IOP‐lowering eye drops from a preoperative mean of 3.0 ± 0.4, to a postoperative mean of 0.8 ± 1.5 (Naito et al., 2017). These changes led to a change in the VF MD slope that went from −0.91 ± 0.57 to −0.32 ± 0.57 dB/year (30–2 VF test, p‐value = 0.0334) and from −0.97 ± 0.70 to −0.09 ± 0.37 dB/year (10–2 VF test, p‐value = 0.0709). They conducted a sub‐analysis to analyse the proportion of eyes with VF MD slope change when divided into postoperative IOP < 10 mmHg (single‐digit IOP group) and postoperative IOP > 10 mmHg (double‐digit IOP group). They concluded that 91.7% of the eyes from the single‐digit IOP group showed a positive change of the VF MD slope, while only 20.0% of the eyes in the double digit IOP group achieved that. Moreover, the group that showed no change in VF MD slope had a significantly higher mean postoperative IOP (11.2 ± 2.4 mmHg vs. 6.8 ± 2.0 mmHg) and a significantly lower mean IOP reduction (21.7 ± 12.5% vs. 50.0 ± 17.3%). The group showing a positive change in VF MD slope also used a lower mean of IOP‐lowering eye drops after surgery in comparison to the other group (0.3 ± 1.2 vs. 2.0 ± 1.6) (Naito et al., 2017).

Consistently, Nakajima et al. showed an IOP reduction of 35.3 ± 19.7%, from a baseline mean IOP of 13.9 ± 3.1 mmHg to a final mean IOP of 9.0 ± 4.6 mmHg, which was accompanied by a reduction of the number of IOP‐lowering eye drops from a preoperative mean of 2.6 ± 0.5 to a postoperative mean of 0.1 ± 0.4 (Nakajima et al., 2021). These changes led to a change in the VF MD slope that went from a mean of −0.52 ± 0.057 dB/year to a mean of −0.34 ± 0.17 (30–2 VF test, p‐value = 0.31) and from a mean of −1.00 ± 0.13 dB/year to a mean of −0.41 ± 0.10 (10–2 VF test, p‐value ≤0.01) (Nakajima et al., 2021).

Further supporting these results, Oie et al. compare the effect of different rates of IOP reduction in NTG progression between three groups: a group of patients who were submitted to trabeculectomy (group 1), a group of patients with an IOP reduction of >15% with prostaglandin analogues (group 2), and a group of patients with an IOP reduction of <15% with prostaglandin analogues (group 3) (Oie et al., 2017). They demonstrated that in group 1 an IOP reduction of 37.6 ± 14.3% was accomplished, with IOP reducing from a mean of 14.7 ± 1.3 mmHg to a mean of 9.1 mmHg after trabeculectomy, leading to a change of the VF MD slope from −0.86 ± 0.51 dB/y to −0.19 ± 0.20 dB/year (p‐value <0.001). Group 2 had a pre‐intervention mean IOP of 14.7 ± 1.5 mmHg, and with an IOP reduction of 20.2 ± 3.8%, went to a mean of 11.7 ± 1.2 mmHg, also leading to a significant, yet less pronounced, change in the VF MD slope (from −0.52 ± 0.37 dB/y to −0.31 ± 0.30, p‐value = 0.019). In contrast, group 3, with an IOP reduction of 8.2 ± 5.2% (from 14.4 ± 1.8 mmHg to 13.2 ± 1.6 mmHg), showed no significant change in the VF MD slope (from −0.40 ± 0.27 dB/y to −0.50 ± 0.65, p‐value = 0.458) (Oie et al., 2017).

Providing additional evidence for these findings, Shigeeda et al. documented a mean IOP of 16.2 ± 1.8 mmHg and a mean VF MD slope of −1.05 (−1.28 to −0.82) preoperatively (Shigeeda et al., 2002). After trabeculectomy, IOP decreased to a mean of 10.8 ± 2.2 mmHg, accounting for an IOP reduction of 33.33 ± 7.73%, and VF MD slope changed to −0.44 (−0.64 to −0.24) (p‐value <0.001) (Shigeeda et al., 2002).

Additionally, the likelihood of VF defect aggravation, based on the VF MD slope, was significantly lower in patients achieving an IOP ≤10 mmHg, as reported by Aoyama et al. (Aoyama et al., 2010). In this study, multiple IOP targets were tested to determine the best IOP target level in NTG patients for VF stability following trabeculectomy. Overall, trabeculectomy resulted in a mean IOP reduction of 38.16% ± 9.21% (from 15.2 ± 1.4 mmHg to 9.4 mmHg). Regarding the eyes with postoperative IOP <12 mmHg, 20.6% showed a VF defect aggravation, contrary to 50.0% of eyes with IOP >12 mmHg (p‐value = 0.1528). For IOP <11 mmHg and IOP >11 mmHg, 17.2% and 45.4% demonstrated VF defect aggravation, respectively (p‐value = 0.1028). As for those with a postoperative IOP <10 mmHg, 8.3% had VF defect aggravation, compared to 50.0% of those with IOP > 10 mmHg (p‐value = 0.0068). Of eyes with a postoperative IOP of <9 mmHg, 11.8% showed VF defect aggravation, compared to 34.8% of those with IOP >9 mmHg (p‐value = 0.1450) (Aoyama et al., 2010).

### 3.3 Quantitative synthesis of results

Eight studies were included in the meta‐analysis, each providing sufficient data to calculate paired mean differences in VF MD slope (dB/year) before and after the intervention. Substantial heterogeneity was observed across studies. The I^2^ statistic was 93.3%, and the between‐study variance (τ^2^) was estimated at 0.0635, indicating considerable inconsistency in effect estimates. As pre‐specified, a random‐effects model was applied to account for this variability. The pooled mean difference was 0.59 dB/year (95% CI: 0.38 to 0.80, *p* < 0.0001), indicating a statistically significant improvement in VF progression following the intervention (Figure 2).

**FIGURE 2:**
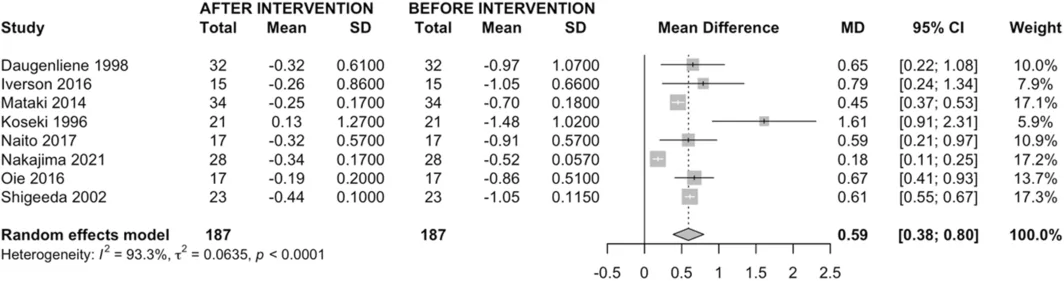
Pooled mean difference in VF MD slope before and after intervention.

Given the high degree of heterogeneity, a leave‐one‐out sensitivity analysis was performed to assess the influence of individual studies on the overall pooled estimate (Figure 3). The pooled mean difference remained statistically significant across all iterations, with values ranging from 0.51 to 0.64 db/year. Most individual exclusions had a minimal impact on the overall estimate and heterogeneity metrics. Excluding the study by Nakajima et al. led to a marked reduction in heterogeneity (I^2^ = 69.1%, τ^2^ = 0.0139), while preserving a consistent effect size (mean difference = 0.61 [95% CI: 0.48 to 0.75]) (Nakajima et al., 2021). This suggests that Nakajima et al. substantially contributed to between‐study variability (Nakajima et al., 2021). A secondary analysis was therefore performed without this study to assess its impact on overall heterogeneity and the robustness of the pooled estimate (Figure 4).mean difference (Nakajima et al., 2021). The pooled estimate of the mean difference increased slightly to 0.61 dB/year (95% CI: 0.48 to 0.75, p < 0.0001), and the heterogeneity decreased markedly, from I^2^ = 93.3% (τ^2^ = 0.0635) to I^2^ = 69.1% (τ^2^ = 0.0139, *p* = 0.0035). This substantial reduction in heterogeneity, without altering the effect size's direction or significance, reinforces the findings' robustness and supports the consistency of the intervention's beneficial impact across the remaining studies.

**FIGURE 3:**
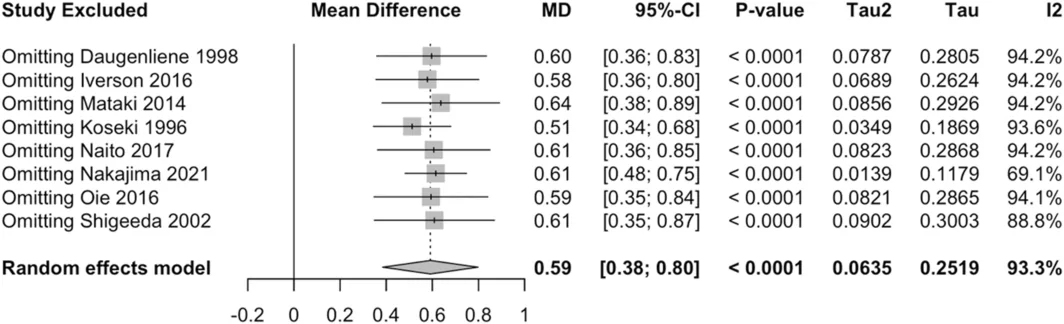
Leave‐one‐out sensitivity analysis.

**FIGURE 4:**
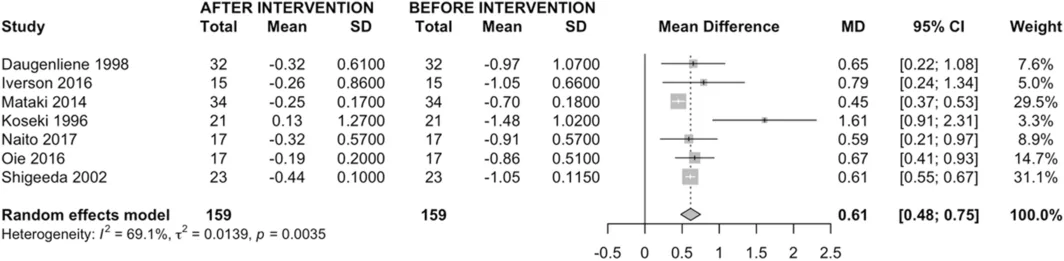
Pooled mean difference in VF MD slope before and after intervention, after the removal of the study by Nakajima et al.

The funnel plot (Figure 5) shows a reasonably symmetrical distribution around the pooled mean difference, with no clear evidence of publication bias. Although Koseki et al. stand out with a larger effect size, the observed heterogeneity is more likely due to clinical or methodological differences than to small‐study effects (Koseki et al., 1997).

**FIGURE 5:**
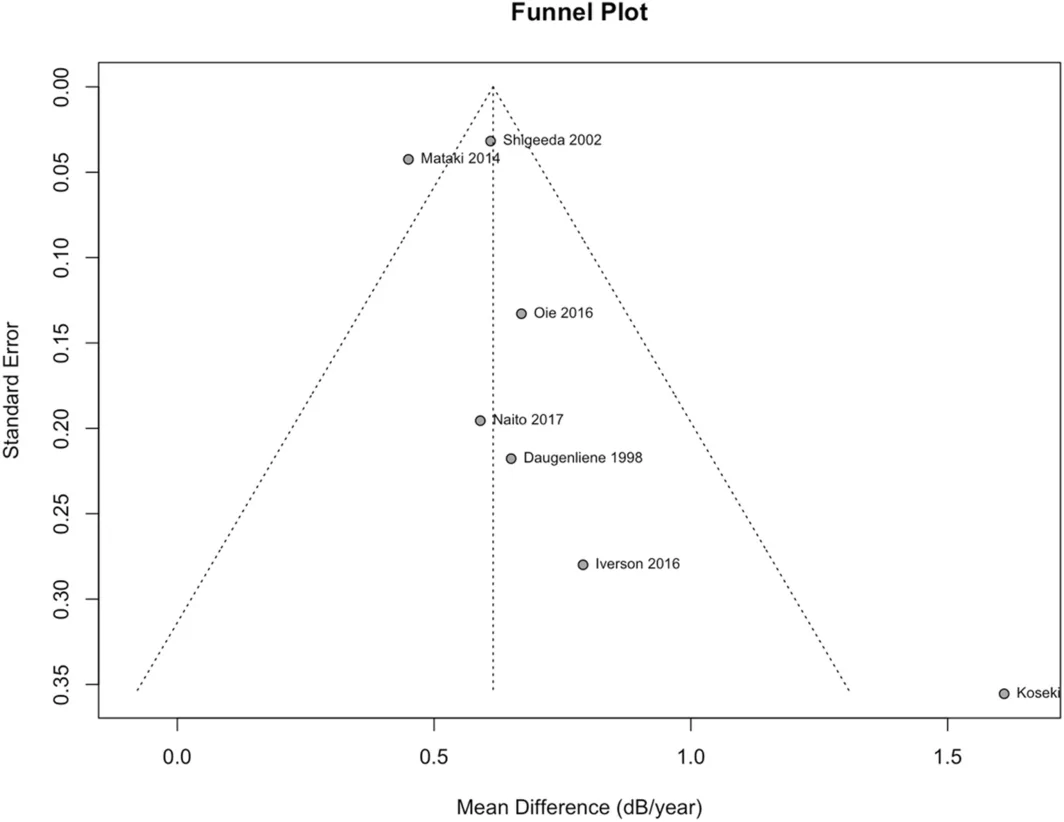
Funnel plot of the studies included in the meta‐analysis.

### 3.4 Sensitivity analysis based on assumed paired correlation

To test the robustness of the results to assumptions about the correlation between pre‐ and post‐intervention values, a sensitivity analysis was conducted using alternative within‐subject correlation coefficients. In addition to the primary model assuming r = 0.5, two further scenarios were tested (r = 0.25 and r = 0.75), in accordance with Cochrane guidance for paired data when correlations are not reported.

Across all three correlation scenarios, the pooled mean difference in VF MD slope remained statistically significant and consistent in magnitude. The estimated effect ranged from 0.62 dB/year (r = 0.25) to 0.67 dB/year (r = 0.75), with overlapping CI and stable direction of effect. As expected, higher correlation assumptions led to slightly narrower standard errors and greater heterogeneity, with I^2^ increasing from 96.1% (r = 0.25) to 99.0% (r = 0.75). These findings suggest that the overall result is robust to reasonable variations in the assumed correlation, and that the beneficial effect of the intervention on VF progression is not dependent on this assumption (see figure, **Supplemental Content** **3**, which demonstrates the stability of the pooled mean difference in VF MD slope (dB/year) and its 95% CI across scenarios and see table, **Supplemental Content** **4**, which demonstrates the effect of different assumed within‐subject correlation values (r) on the pooled mean difference in VF MD slope (dB/year).

### 3.5 Is it safe to further reduce the IOP to single‐digits levels?

The safety of lowering IOP to single‐digit levels in eyes with progressive NTG was assessed through the incidence of post‐intervention complications and changes in visual acuity. Only three studies (33.3%) provided data on these parameters (Aoyama et al., 2010; Iverson et al., 2016; Naito et al., 2017). Overall, 60% of eyes in Iverson et al. and 70.6% in Naito et al. experienced at least one early or late postoperative complication (Iverson et al., 2016, Naito et al., 2017).

Hypotony and related complications (maculopathy and choroidal detachment) were the most prevalent complications reported across the three studies (Aoyama et al., 2010, Iverson et al., 2016, Naito et al., 2017). Iverson et al. documented the occurrence of hypotony in 5 out of 15 (33.3%) eyes as an early complication and in 6 out of 15 eyes (40%) as a late complication (Iverson et al., 2016). The baseline and postoperative VA in hypotonus eyes were compared, showing that pre‐trabeculectomy VA ranged from 20/20 to 20/40, while post‐trabeculectomy VA ranged from 20/20 to 20/60, and no patient experienced a VA decrease of more than two Snellen lines. Additionally, postoperative best‐corrected visual acuity (BCVA) (log MAR) was compared between eyes that developed hypotony and those that did not, revealing no significant difference (0.20 ± 0.24 vs. 0.18 ± 0.32, respectively). Naito et al. observed transient hypotony in 9 out of 17 eyes (52.9%), drawing attention to its frequent occurrence and reported a VA decline by ≥0.1 unit in 3 eyes (17.6%) (Naito et al., 2017). Across the nine included studies, data on the long‐term effect of hypotony on visual acuity were limited. Nevertheless, available evidence suggests that, despite the relatively high incidence of hypotony, most eyes maintained stable visual acuity over long‐term follow‐up, with only a minority experiencing mild visual acuity decline.

Another hypotony‐related complication verified in these studies was choroidal detachment. This condition was presented as an early complication by Iverson et al., occurring in 1 of the 15 eyes included (6.7%) (Iverson et al., 2016). Naito et al., with the highest prevalence of this complication, observed this situation in 4 out of 17 eyes (23.5%), also as an early complication (Naito et al., 2017). Other complications, such as hypotony maculopathy, were observed in a single case out of 40 included by Aoyama et al. (2.5%) (Aoyama et al., 2010). This complication was also reported by Iverson et al. as a late complication in 1 out of 15 eyes (6.7%). However, no surgical intervention was needed in that eye, based on unchanged VA levels, before and after the intervention. Similarly, and with the highest prevalence, Naito et al. documented this event in 3 out of 17 cases (17.6%), leading to a decline in VA of >0.1 unit, also as a late complication (Naito et al., 2017).

Following hypotony, hyphema was one of the most frequently reported complications across the reviewed studies, with 2 out of 15 eyes (13.33%) and 4 out of 17 eyes (23.5%) verifying this complication in Iverson et al. and Naito e al., respectively (Iverson et al., 2016, Naito et al., 2017).

### 3.6 How easy is it to achieve and maintain single‐digit IOP values?

The capacity to achieve and sustain single‐digit IOP levels is a key factor in the management of progressive NTG. Three of the included studies (33.3%) evaluated the long‐term achievability of IOP reduction to single‐digit levels, also providing data on the durability of this outcome (Aoyama et al., 2010; Iverson et al., 2016; Naito et al., 2017). Aoyama et al. reported that throughout the follow‐up period (12 ± 2.5 years), a postoperative IOP of <10 mmHg was attained and sustained by 60.0% of cases (Aoyama et al., 2010). Iverson et al. analysed the overall cumulative probability of successfully achieving a single‐digit IOP target during the follow‐up period, which was 87%, 80%, 80% and 66% at 1, 2, 3 and 4 years of follow‐up, respectively (Iverson et al., 2016). Naito et al. reported that 70.6% of the total number of eyes achieved an IOP < 10 mmHg (Naito et al., 2017).

## 4 DISCUSSION

NTG is a progressive optic neuropathy characterized by VF deterioration despite IOP levels within the statistically normal range. Given that IOP remains the only modifiable risk factor in glaucoma management, lowering IOP is the primary therapeutic strategy for slowing disease progression. However, a significant subset of NTG patients continues to experience VF deterioration despite optimized pharmacological treatment, raising the question of whether more aggressive IOP reduction, specifically to single‐digit levels, offers additional benefits.

Our findings indicate that achieving single‐digit IOP levels (or IOP ≤10 mmHg) in progressive NTG patients is significantly correlated with a deceleration of glaucoma progression. All studies opted to perform a trabeculectomy with an antimetabolite. Across all the studies analysed, patients who reached IOP levels ≤10 mmHg demonstrated significant change in their VF parameters, particularly the VF MD slope (pooled mean difference: 0.59 dB/year), as well as a decreased probability of VF defect aggravation. Additionally, the mean number of IOP‐lowering medications was markedly reduced following interventions, which may also benefit the patients' quality of life by improving their ocular surface (Kolko et al., 2023). The results of this systematic review and meta‐analysis are consistent with the current understanding of NTG pathophysiology and therapeutic response and are therefore expected both in direction and in magnitude. The observed results align with the well‐established notion that additional decreases in IOP can have a positive impact on disease progression in NTG (Girkin et al., 2008; Razeghinejad & Lee, 2019).

Despite achieving and maintaining postoperative mean IOP levels below 10 mmHg, VF progression was not completely halted, with a mean postoperative MD slope of −0.25 ± 0.16 dB/year. Although this rate represents a marked improvement compared to preoperative progression, it still seems to be significantly different from zero, indicating residual disease activity even under single‐digit IOP levels. This finding is consistent with previous longitudinal data. Sakata et al. reported a mean MD slope of −0.33 dB/year (95% CI −0.22 to −0.44) over a 5‐year follow‐up in untreated NTG eyes with a mean IOP of 12.4 ± 1.2 mmHg. Notably, despite the substantially lower mean (treated) IOP achieved in the present review (9.8 ± 1.6 mmHg), the postoperative rate of progression appears broadly comparable (Sakata, 2019). This may be explained by the fact that VF deterioration in NTG is likely also influenced by additional IOP‐independent mechanisms. Importantly, surgical intervention may confer benefits beyond mean IOP reduction, particularly through a reduction in short‐ and long‐term IOP fluctuations, which have been increasingly recognized as contributors to glaucoma progression. Recent evidence suggests that both mean IOP and IOP variability play a significant role in disease progression, and that trabeculectomy may provide a more stable IOP profile compared to medical therapy alone. This stabilization may partly explain the favourable change in progression rate observed after surgery, even if progression is not entirely eliminated (Ruparelia, 2025).

Our findings are consistent with previous evidence. Lai et al. analysed and summarized the results of seven studies regarding the impact of trabeculectomy with antimetabolites on VF, IOP and antiglaucoma medications in progressive NTG. They included a total of 166 eyes and demonstrated a significant decrease in IOP after trabeculectomy (pooled mean difference: −5.54 mmHg, 95% CI: −6.02 to −5.06, I2 = 0%) that was associated with a significant change in the VF MD slope (pooled mean difference: 0.54 dB/year, 95% CI: 0.40 to 0.67, I2 = 9%) (Lai et al., 2022).

When evaluating the long‐term efficacy of new treatment strategies, a crucial aspect to investigate is the achievability and sustainability of such a strategy. Some of the studies included in this review demonstrated that single‐digit IOP levels are not only achievable through surgical interventions but also a sustainable target over time. This finding is particularly relevant as treatment success is not only determined by the ability to lower IOP to single‐digit levels but also by its capacity to sustain this reduction over time. For this reason, we suggest that future evidence should focus on both the efficacy of IOP reduction and the sustainability of this approach.

As described before, achieving single‐digit levels of IOP was associated with therapeutic benefits and a slower rate of VF progression. However, the balance between efficacy and safety must be weighed since aggressively lowering IOP is also associated with an increased risk of complications, mainly those related to hypotony. Only three studies (33.3%) reported post‐intervention complications. Ideally, all studies assessing the efficacy of an intervention should also evaluate its safety, since treatment implementation in glaucoma management requires both efficacy and safety. Furthermore, studies reporting hypotony and hypotony‐related complications should distinguish between numerical hypotony (IOP <6 mmHg without associated complications) and clinically significant hypotony, as recommended by the European Glaucoma Society (EGS) (Abegao Pinto et al., 2023). Clinically significant hypotony is defined as IOP <6 mmHg associated with choroidal detachment and/or hypotony maculopathy and/or anterior chamber (AC) depth reduction, or IOP <6 mmHg requiring surgical intervention to reverse hypotony‐related complications. In the included studies, hypotony maculopathy was observed in up to 17.6% of cases, with some patients experiencing a decline in visual acuity. Choroidal detachment was also frequently reported (ranging from 6.7% to 23.5%), particularly in early postoperative stages. While studies report that most hypotony cases resolved spontaneously, in some cases, visual function was permanently affected. These complications highlight the difficulty of accomplishing an optimal balance between achieving a sufficiently low IOP to slow down glaucoma progression and avoiding significant complications. Clinicians should consider more caution when dealing with eyes that are more prone to hypotony, such as high myopes or vitrectomized eyes. Frequent follow‐up visits in progressing NTG patients after trabeculectomy with antimetabolite drugs are suggested.

Trabeculectomy has proven to be an effective and safe surgical strategy for obtaining significant IOP reduction in NTG patients. The findings of this systematic review suggest that trabeculectomy can successfully lower IOP to single‐digit levels and sustain these reductions over time. These results support the role of trabeculectomy as a crucial treatment modality for progressive NTG patients who fail to respond to medical therapy. Trabeculectomy has been identified as the surgical option that allows the lowest postoperative absolute IOP values to be reached (Gedde et al., 2023). Nonetheless, there were no comparative studies with other surgical options that would have allowed us to assess if other options would also be effective.

The results of our systematic review may have important implications for the future management of NTG. By demonstrating the potential benefits of more aggressive IOP reduction, this study provides evidence supporting the consideration of surgical intervention to achieve single digit IOP levels at an earlier stage in the disease progression. Furthermore, highlighting the balance between the efficacy and safety of this therapeutic approach may help guide clinicians in adopting individualized treatment strategies, contributing to favourable outcomes and preservation of visual function in NTG patients.

Several limitations of this study should be acknowledged. First, only observational studies were included, which limits the level of evidence, as RCTs were not found. Second, clinical and methodological heterogeneity among the included studies should be considered when interpreting the findings, since relevant heterogeneity was identified within our meta‐analysis. Third, as most of the included studies were conducted in Japan, where NTG prevalence is highest, further research from diverse populations is needed to validate these results in broader clinical settings. Finally, although the intended target in our review and in many studies is single‐digit IOP, the cut‐off is frequently defined as IOP ≤10 mmHg. Moreover, only two studies reported mean postintervention IOP above 10 mmHg, and two additional subgroups from one study also remained above this threshold. However, those subgroups were treated with prostaglandin analogues rather than surgical intervention. Lastly, an additional methodological limitation that should be considered is the potential effect of regression to the mean. In clinical practice, surgical intervention is more likely to be offered to eyes with faster rates of VF deterioration, which may result in an apparent improvement in postoperative VF slopes even in the absence of a true causal treatment effect. As a result, part of the observed reduction in VF progression after surgery may reflect a statistical phenomenon inherent to before‐after study designs without a concurrent control group, potentially leading to an overestimation of the intervention's effect size. Therefore, although the direction and consistency of the findings across studies support a beneficial association between aggressive IOP reduction and slower VF progression, causal inference should be made with caution and future studies using controlled comparative designs or analytical approaches that better account for baseline progression rates are warranted.

## 5 CONCLUSIONS

Further reduction of IOP to single‐digit levels (or IOP ≤10 mmHg) in treated NTG patients with glaucoma progression is associated with a slowdown in the VF progression rate. Trabeculectomy is an effective and safe strategy to achieve these targets. However, significant hypotony‐related complications may occur. Thus, the balance between efficacy and safety should be evaluated on a case‐by‐case basis to optimize individual outcomes.

## Supporting information


**Supplemental Content 1.** Queries used for searching MEDLINE/PubMed, Web of Science and Scopus.

**Supplemental Content 2.** Quality Assessment with NIH Quality Assessment Tool for Before‐After (Pre‐Post) Studies with No Control Group.

**Supplemental Content 3.** Graphical representation of the sensitivity analysis for different assumed within‐subject correlation values (r), illustrating the stability of the pooled mean difference in VF MD slope (dB/year) and its 95% confidence intervals across scenarios.

**Supplemental Content 4**. Sensitivity analysis showing the effect of different assumed within‐subject correlation values (r) on the pooled mean difference in VF MD slope (dB/year), including corresponding 95% confidence intervals and heterogeneity estimates (I^2^ and τ^2^).
