# Supplementary material for: The efficacy and safety of reducing intraocular pressure to single‐digit levels in Normal‐tension glaucoma: A systematic review and meta‐analysis
Source: Acta Ophthalmol. 2026 Mar 20;104(7):720–30. doi: 10.1111/aos.70110 (PMC13651934; doi:10.1111/aos.70110)
Supplement: Supplementary file 1 [file AOS-104-720-s001.docx]

**Supplemental content 1.** Search Query per Database

| Database | Search Query | Number of results |
| --- | --- | --- |
| PubMed  29/04/2024 | ("Low pressure glaucoma"[Text Word] OR "low tension glaucoma"[Text Word] OR "Normotensive glaucoma"[Text Word] OR "Normal tension glaucoma"[Text Word] OR "Normal pressure glaucoma"[Text Word] OR "low tension glaucoma"[MeSH Terms]) AND ("single digit IOP"[Text Word] OR "IOP<10"[Text Word] OR "IOP≤10"[Text Word] OR (Progression[Text Word] AND <10[Text Word]) OR (progressive*[Text Word] AND <10[Text Word]) OR (Progression[Text Word] AND ≤10[Text Word]) OR (progressive*[Text Word] AND ≤10[Text Word])) | 136 |
| Scopus  29/04/2024 | ( TITLE-ABS-KEY ( "Low pressure glaucoma" OR "Low tension glaucoma" OR "Normotensive glaucoma" OR "Normal tension glaucoma" OR "Normal pressure glaucoma" ) AND TITLE-ABS-KEY ( "single digit" OR "IOP<10" OR "IOP≤10" OR ( progression AND <10 ) OR ( progressive* AND <10 ) OR ( progression AND ≤10 ) OR ( progressive* AND ≤10 ) ) ) | 96 |
| Web of Science  29/04/2024 | (TS=("Low pressure glaucoma" OR "Low tension glaucoma" OR "Normotensive glaucoma" OR "Normal tension glaucoma" OR "Normal pressure glaucoma" )) AND TS=("single digit" OR "IOP<10" OR "IOP≤10" OR ( progression AND <10 ) OR ( progressive* AND <10 ) OR ( progression AND ≤ 10 ) OR ( progressive* AND ≤ 10 ) ) | 470 |
