# Supplementary material for: The efficacy and safety of reducing intraocular pressure to single‐digit levels in Normal‐tension glaucoma: A systematic review and meta‐analysis
Source: Acta Ophthalmol. 2026 Mar 20;104(7):720–30. doi: 10.1111/aos.70110 (PMC13651934; doi:10.1111/aos.70110)
Supplement: Supplementary file 2 [file AOS-104-720-s002.docx]

| **Correlation (r)** | **MD (95% CI)** | **I² (%)** | **Tau²** |
| --- | --- | --- | --- |
| 0.25 | 0.616 (0.395-0.836) | 96.1 | 0.0789 |
| 0.5 | 0.643 (0.403-0.884) | 97.8 | 0.1032 |
| 0.75 | 0.669 (0.406-0.933) | 99.0 | 0.1342 |

**Supplemental content 4.** Sensitivity analysis showing the effect of different assumed within-subject correlation values (r) on the pooled mean difference in VF MD slope (dB/year), including corresponding 95% confidence intervals and heterogeneity estimates (I² and Tau²).

Values of r = 0.25, 0.5, and 0.75 were selected to explore the impact of different assumptions about within-subject correlation on the variance of the paired mean differences.
