# Supplementary material for: The efficacy and safety of reducing intraocular pressure to single‐digit levels in Normal‐tension glaucoma: A systematic review and meta‐analysis
Source: Acta Ophthalmol. 2026 Mar 20;104(7):720–30. doi: 10.1111/aos.70110 (PMC13651934; doi:10.1111/aos.70110)
Supplement: Supplementary file 3 [file AOS-104-720-s003.docx]

**Supplemental Content 3**. jpeg Graphical representation of the sensitivity analysis for different assumed within-subject correlation values (r), illustrating the stability of the pooled mean difference in VF MD slope (dB/year) and its 95% confidence intervals across scenarios.
