# Supplementary material for: The efficacy and safety of reducing intraocular pressure to single‐digit levels in Normal‐tension glaucoma: A systematic review and meta‐analysis
Source: Acta Ophthalmol. 2026 Mar 20;104(7):720–30. doi: 10.1111/aos.70110 (PMC13651934; doi:10.1111/aos.70110)
Supplement: Supplementary file 4 [file AOS-104-720-s004.docx]

| Criteria | Aoyama  2010 | Daugeliene  1998 | Iverson 2016 | Koseki 1996 | Mataki 2014 | Naito 2017 | nakajima 2021 | Oie  2016 | Shigeeda 2002 |
| --- | --- | --- | --- | --- | --- | --- | --- | --- | --- |
| 1 | Yes | Yes | Yes | Yes | Yes | Yes | Yes | Yes | Yes |
| 2 | Yes | Yes | Yes | Yes | Yes | Yes | Yes | Yes | Yes |
| 3 | Yes | Yes | Yes | Yes | Yes | Yes | Yes | Yes | Yes |
| 4 | No | No | No | No | No | No | No | Yes | No |
| 5 | Yes | Yes | Yes | Yes | Yes | Yes | Yes | Yes | Yes |
| 6 | Yes | Yes | Yes | Yes | Yes | Yes | Yes | Yes | Yes |
| 7 | Yes | Yes | Yes | Yes | Yes | Yes | Yes | Yes | Yes |
| 8 | NR | NR | NR | NR | NR | NR | NR | NR | NR |
| 9 | Yes | Yes | Yes | Yes | Yes | Yes | Yes | Yes | Yes |
| 10 | Yes | Yes | Yes | Yes | Yes | Yes | Yes | Yes | Yes |
| 11 | Yes | Yes | Yes | Yes | Yes | Yes | Yes | Yes | Yes |
| 12 | Yes | Yes | Yes | Yes | Yes | Yes | Yes | Yes | Yes |
| rating | **GOOD** | **GOOD** | **GOOD** | **GOOD** | **GOOD** | **GOOD** | **GOOD** | **GOOD** | **GOOD** |

**Supplemental content 2.** Quality Assessment with NHLBI Quality Assessment Tool for Before-After (Pre-Post) Studies with No Control Group.

1. Was the study question or objective clearly stated?

2. Were eligibility/selection criteria for the study population prespecified and clearly described?

3. Were the participants in the study representative of those who would be eligible for the test/service/intervention in the general or clinical population of interest?

4. Were all eligible participants that met the prespecified entry criteria enrolled?

5. Was the sample size sufficiently large to provide confidence in the findings?

6. Was the test/service/intervention clearly described and delivered consistently across the study population?

7. Were the outcome measures prespecified, clearly defined, valid, reliable, and assessed consistently across all study participants?

8. Were the people assessing the outcomes blinded to the participants' exposures/interventions?

9. Was the loss to follow-up after baseline 20% or less? Were those lost to follow-up accounted for in the analysis?

10. Did the statistical methods examine changes in outcome measures from before to after the intervention? Were statistical tests done that provided p values for the pre-to-post changes?

11. Were outcome measures of interest taken multiple times before the intervention and multiple times after the intervention (i.e., did they use an interrupted time-series design)?

12. If the intervention was conducted at a group level (e.g., a whole hospital, a community, etc.) did the statistical analysis take into account the use of individual-level data to determine effects at the group level?
